# Comparison of frequency-domain and continuous-wave near-infrared spectroscopy devices during the immediate transition

**DOI:** 10.1186/s12887-020-1987-4

**Published:** 2020-02-28

**Authors:** Tanja van Essen, Tom G. Goos, Liza van Ballegooijen, Gerhard Pichler, Berndt Urlesberger, Irwin K. M. Reiss, Rogier C. J. de Jonge

**Affiliations:** 1 Department of Pediatrics, Division of Neonatology, Erasmus MC - Sophia Children’s Hospital, Dr. Molewaterplein 40, 3015GD Rotterdam, The Netherlands; 2 Department of Biomechanical Engineering, Delft University of Technology, Mekelweg 5, 2628CD Delft, The Netherlands; 3 Research Unit for Neonatal Micro- and Macrocirculation, Department of Pediatrics and Adolescent Medicine, Medical University of Graz, Auenbruggerplatz 2, 8036 Graz, Austria; 4 Division of Neonatology, Department of Pediatrics and Adolescent Medicine, Medical University of Graz, Auenbruggerplatz 2, 8036 Graz, Austria; 5 Pediatric Intensive Care Unit, Department of Pediatrics and Pediatric Surgery, Erasmus MC - Sophia Children’s Hospital, Dr. Molewaterplein 40, 3015GD Rotterdam, The Netherlands

**Keywords:** Near-infrared spectroscopy, Continuous-wave, Frequency-domain, Transition, Preterm neonate

## Abstract

**Background:**

Non-invasive monitoring of cerebral tissue oxygen saturation (rcSO_2_) during transition is of growing interest. Different near-infrared spectroscopy (NIRS) techniques have been developed to measure rcSO_2_. We compared rcSO_2_ values during the immediate transition in preterm neonates measured with frequency-domain NIRS (FD-NIRS) with those measured with continuous-wave NIRS (CW-NIRS) devices in prospective observational studies.

**Methods:**

We compared rcSO_2_ values measured with an FD-NIRS device during the first 15 min after birth in neonates with a gestational age ≥ 30 weeks but < 37 weeks born at the Erasmus MC- Sophia Children’s Hospital, Rotterdam, the Netherlands, with similar values measured with a CW-NIRS device in neonates born at the Medical University of Graz, Austria. Mixed models were used to adjust for repeated rcSO_2_ measurements, with fixed effects for time (non-linear), device, respiratory support and the interaction of device and respiratory support with time. Additionally, parameters such as total haemoglobin concentration and oxygenated and deoxygenated haemoglobin concentrations measured by FD-NIRS were analysed.

**Results:**

Thirty-eight FD-NIRS measurements were compared with 58 CW-NIRS measurements. The FD-NIRS rcSO_2_ values were consistently higher than the CW-NIRS rcSO_2_ values in the first 12 min, irrespective of respiratory support. After adjustment for respiratory support, the time-dependent trend in rcSO_2_ differed significantly between techniques (*p* < 0.01).

**Conclusion:**

As cerebral saturation measured with the FD-NIRS device differed significantly from that measured with the CW-NIRS device, differences in absolute values need to be interpreted with care. Although FD-NIRS devices have technical advantages over CW-NIRS devices, FD-NIRS devices may overestimate true cerebral oxygenation and their benefits might not outweigh the usability of the more clinically viable CW-NIRS devices.

## Background

**Fig. 1:**
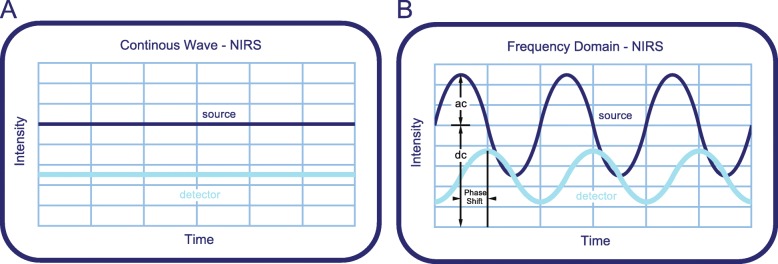
Graphic representation of the emitted and detected light beam for the CW-NIRS and FD-NIRS techniques. The dark blue line represents the light entering the tissue; the light blue line represents the light that is detected at the detector. **a**, Intensity of emitted and detected light of the CW-NIRS technique. **b**, Intensity of the modulated light beam and measured parameters. Image adapted from OxiplexTS™ brochure with the courtesy of ISS, Champaign, Illinois, USA

Poor cerebral perfusion and fluctuations in cerebral oxygenation can adversely affect brain development [1–3]. Regional tissue oxygenation can be continuously monitored with near-infrared spectroscopy (NIRS). The use of this non-invasive technique in neonates allows for the deployment of interventions to stabilize or improve cerebral oxygenation and perfusion [1, 4]. Various NIRS-based measurement techniques, devices and sensors have been developed to monitor cerebral oxygenation. Most of the clinically used NIRS devices make use of continuous-wave (CW) light sources, which emit light with a constant intensity (Fig. 1a). CW-NIRS calculates the oxygen saturation from the measured absorption without the possibility of calculating the absolute oxygenated and deoxygenated haemoglobin concentrations. This technique has great value in monitoring the dynamics of cerebral tissue oxygenation but lacks in providing accurate absolute oxygenation estimates [5].

Another NIRS technique, frequency-domain (FD) NIRS, allows for the determination of absolute values of haemoglobin concentration by modulating the intensity of the emitted light by a sinusoidal function. This results in amplitude of modulation (AC), average intensity (DC) and phase-shift (Fig. 1b) measurements, from which the absolute absorption and scattering coefficients are determined. The phase shift represents the delay between the emitted and detected waves of light and changes with the optical path length of the light through the tissue. From the absorption and scattering coefficients, absolute haemoglobin concentrations and subsequently regional cerebral oxygen saturation (rcSO_2_) are calculated. Measurements of absolute concentrations of haemoglobin may improve the interpretation of brain haemodynamics during various clinical settings and allow for within- and between-patient comparisons. While the CW-NIRS technique has been extensively evaluated in the clinical setting, including the transition after birth [6], bedside FD-NIRS devices are not yet available, and the technique has yet to be evaluated properly in a clinical setting.

The aim of the present study was to compare the results of CW-NIRS and FD-NIRS monitoring devices in preterm neonates immediately after birth. If, in theory, both devices reflect true tissue oxygenation, we hypothesized that the cerebral oxygenation values measured with each device would be similar in absolute values as well as in trend during the transition after birth. As cerebral saturation gradually increases during the transition phase after birth, the measurement techniques can be compared over the full dynamic range of tissue oxygenation. In addition, data obtained with the FD-NIRS device might help us to understand the physiological changes that occur immediately after birth.

## Methods

In this study, data from prospective observational studies performed at the Erasmus MC - Sophia Children’s Hospital, Rotterdam, the Netherlands, and the Medical University of Graz, Graz, Austria, were compared. Those studies concerned neonates with a gestational age ≥ 30 weeks but < 37 weeks, monitored during the first 15 min of life with an FD-NIRS device in Rotterdam, and with a CW-NIRS device in Graz.

### FD-NIRS

FD-NIRS measurements were performed in newborn neonates between May 2015 and October 2017, delivered either vaginally or by caesarean section. Neonates with suspected congenital or chromosomal anomalies were excluded from the analyses. The local Medical Ethics Review Board waived approval (argument: “Medical Research in Human Subjects Act does not apply to this research proposal”; MEC-2011-415).

FD-NIRS measurements were performed using the OxiplexTS™ (ISS, Inc., Champaign, IL, USA) with the Infant Flexible Sensor, containing one detector and four emitter positions with two emitters each (eight in total). Emitter-detector distances on this device range from 1.5 cm to 4.0 cm. The device uses near-infrared light at two different wavelengths: 684 nm and 828 nm. Continuous FD-NIRS measurement data were collected over a maximum of 15 min, with a sampling rate of 0.5 s (2 Hz).

In addition to the preductal arterial oxygen saturation (SpO_2_) measurement used as standard of care, postductal SpO_2_ and heart rate (HR) were measured with pulse oximetry (Masimo Radical-7, Irvine, CA, USA). Any respiratory support was provided using a T-piece resuscitator (Neopuff, Fisher & Paykel Healthcare, Auckland, New Zealand).

### CW-NIRS

For comparison, rcSO_2_ values obtained with a CW-NIRS device were provided by the Graz University Medical Centre. The data were obtained in prospective observational studies approved by the Regional Committee on Biomedical Research Ethics at the Medical University of Graz (EK- number: 19-291ex07/08, 23-403ex10/11, 27-465ex14/15). Written informed consent was obtained from the parents before the birth of the infant. All included neonates had been delivered by elective caesarean section. The CW-NIRS device used was the INVOS 5100C Cerebral/Somatic Oximeter (Medtronic, Minneapolis, MN, USA). Selected data have been published previously; methods and data acquisition are described in more detail in these publications [7, 8].

### FD-NIRS data acquisition

A sensor calibration procedure was carried out before every measurement. The measurement time started when the umbilical cord was clamped, as this was clinical practice in both centres. Further, in both centres, as per the local protocol at the time the study was conducted, immediate cord clamping was the standard procedure and was performed before 30 s. The FD-NIRS sensor was placed on the baby’s left frontotemporal forehead. A pulse oximeter was placed postductally on the baby’s left foot. Upon signs of respiratory distress, supplemental oxygen and positive end-expiratory pressure (PEEP) therapy or ventilation were applied according to the local protocol. Physicians were blinded to the rcSO_2_ and SpO_2_ measurements. In contrast to the data acquisition method applied in Graz, where all neonates were monitored during the full first 15 min after birth, in Rotterdam, we were obligated by the Medical Ethics Review Board to stop the measurements once routine medical care had been completed and the baby was stable before the completion of the first 15 min after birth.

In addition to rcSO_2_, the FD-NIRS device provides absolute values of the total haemoglobin concentration (THb), oxygenated (O_2_Hb) and deoxygenated haemoglobin (HHb) concentration. To account for the displacement of the sensor, during postprocessing of the FD-NIRS data, measurements were discarded when the AC was below 1, the DC was below 10 or when values were considered non-physiological (values below 0 or above 100). Afterwards, the data were averaged over six seconds.

### Phase shift

To compare the CW-NIRS device and the FD-NIRS device, we evaluated the effect of the phase shift on the FD-NIRS measurement. Median (interquartile range; IQR) phase shifts are reported, and the effect of the change of phases over time for both wavelengths on rcSO_2_ was evaluated and presented for a single FD-NIRS measurement. All the phase-shift pairs that occurred during this measurement were used to recalculate a matrix of possible cerebral saturations. The percentiles of these recalculated cerebral saturations for all occurring phase-shift pairs were compared to the original raw cerebral saturation data.

### Statistics

Categorical variables are presented as numbers (%); continuous variables are presented as medians (IQRs). Demographics were compared using Fisher’s exact test for categorical data and the Mann-Whitney U test for continuous data. A per-minute analysis was performed for the rcSO_2_ values, comparing FD-NIRS and CW-NIRS data. The first minute was excluded from the analyses to account for sensor placement. For comparison between devices, data were stratified for the need for respiratory support [7].

To adjust for the repeated rcSO_2_ measurements, we used mixed models to analyse the course of rcSO_2_, SpO_2_, THb, O_2_Hb, and HHb over time. The following fixed effects were considered in the model using backwards selection: the need for respiratory support, delivery method, gestational age, whether the neonate was small for gestational age (dichotomous) and sex. For the random effects, the use of random intercept and slopes were evaluated. To take into account non-linearity in the relation between time and rcSO_2_, SpO_2_, THb, O_2_Hb, and HHb, we explored the use of splines and quadratic terms for time. The final model used a mixed model with fixed effects for (non-linear) time, measurement technique (only for rcSO_2_ and SpO_2_) and respiratory support and a random intercept and slope as random effects. To account for non-linearity, a natural spline with 2 knots for time fit the data best in all mixed models. The results are presented as effect plots of the estimates and their 95% confidence intervals. A two-sided *P*-value of < 0.05 was considered statistically significant. Statistical analyses were performed using the computing environment R (v3.4.1) [9].

## Results

Of 39 eligible neonates in Rotterdam, thirty-eight were included and measured with FD-NIRS. One neonate was diagnosed with ventriculomegaly and therefore excluded. Data from 58 neonates measured with CW-NIRS were available from the Medical University of Graz. Four neonates in Graz had been intubated and were therefore excluded. Table 1 summarizes demographic and clinical data. The groups significantly differed in gestational age and therefore in birthweight and head circumference. After adjustment for gestational age according to Fenton et al. [10], the differences in birthweight and head circumference were not statistically significant. Thirty-four (59%) neonates from the CW-NIRS group and 25 (66%) from the FD-NIRS group needed respiratory support during the first 15 min after birth (*p* = 0.62).

**Table 1:** Demographics and clinical data

	CW-NIRS (*n* = 58)	FD-NIRS (*n* = 38)	*P*-value
Female sex	30 (52%)	14 (37%)	0.22
Gestational age (weeks)	34 ^0^/_7_ [32 ^4^/_7_–35 ^6^/_7_]	32 ^2^/_7_ [31 ^4^/_7_–34 ^1^/_7_]	< 0.01
Birthweight (g)	2066 [1172–2366]	1763 [1480–2011]	0.01
Birthweight for age *SDS*^a^	−0.3 [− 1.0–0.2]	−0.2 [− 0.8–0.6]	0.50
Head circumference (cm)	32.0 [31.0–33.0]	29.5 [28.0–31.8]	< 0.01
Head circumference for age *SDS*^b^	0.0 [0.0–1.0]	0.4 [− 0.8–0.9]	0.91
Arterial umbilical cord pH	7.31 [7.28–7.34]	7.29 [7.26–7.34]	0.39
Apgar			
Minute 1	8.5 [8–9]	8 [7–8]	0.02
Minute 5	9 [8–10]	9 [8–9]	0.12
Minute 10	10 [9–10]	9 [9–10]	0.25
Caesarean section	58 (100%)	27 (71%)	< 0.01
Respiratory support	34 (59%)	25 (66%)	0.62
Reanimation^1^	2 (3%)	0 (0%)	0.39
SGA^2^	7 (12%)	7 (18%)	0.57

Values are median [IQR] or n (%); *SGA* Small for gestational age. ^a^ Birthweight-for-age SDS and ^b^Head circumference-for-age SDS were calculated according to Fenton et al. (ref) ^1^unknown in *n* = 1 from CW-NIRS group; ^2^ unknown in *n* = 1 from CW-NIRS group

### Comparison

**Fig. 2:**
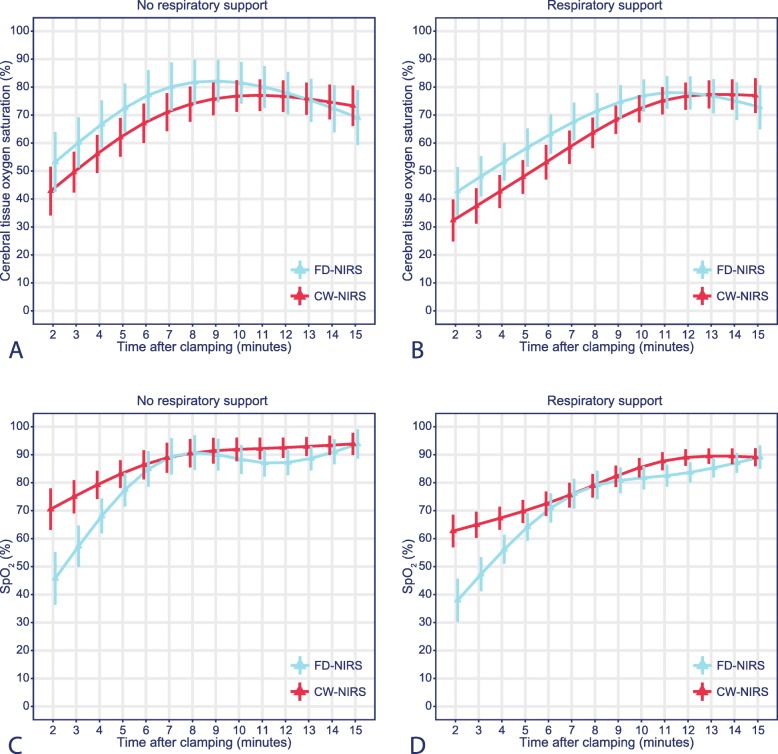
Effect plots of rcSO_2_ and SpO_2_ stratified by the need for respiratory support Symbols represent the estimated means of rcSO_2_ and SpO_2_ with associated 95% confidence intervals for all difference time points based on a mixed model including (non-linear) time, technique, respiratory support and their interaction with time. **a**, Effect plot of rcSO_2_ for neonates not requiring respiratory support. **b**, Effect plot of rcSO_2_ for neonates requiring respiratory support. **c**, Effect plot of SpO_2_ for neonates not requiring respiratory support. **d**, Effect plot of SpO_2_ for neonates requiring respiratory support.

The effect plots of the estimated means stratified for the need for respiratory support are shown for both the CW-NIRS and FD-NIRS measurements (Fig. 2a and b). In the first 12 min, the rcSO_2_ values for the FD-NIRS device were consistently higher than those for the CW-NIRS device. After adjustment for respiratory support, the overall trend of rcSO_2_ over time differed significantly between the devices (*p* < 0.01). Irrespective of the device used, the need for respiratory support significantly influenced cerebral oxygenation (*p* < 0.01).

### Phase shift

**Fig. 3:**
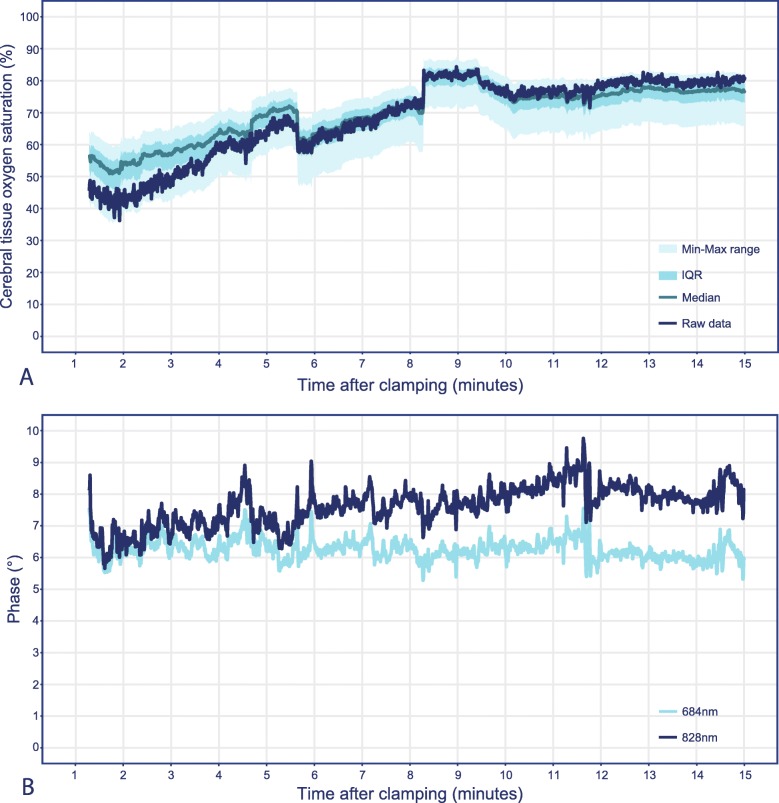
Effect of variance in phase-shift occurrences on cerebral saturation during a single frequency-domain measurement. **a,** Raw data of a single cerebral saturation measurement (dark blue line) and percentiles based on the recalculation of the cerebral saturation measurements based on all phase-shift pair occurrences (light blue ranges). **b,** Raw phase-shift values for 684 nm and 828 nm wavelengths

The raw cerebral saturation values of a single measurement are shown in dark blue in Fig. 3a, with percentiles of recalculated cerebral saturation values based on all occurring phase-shift pairs. Additionally, the raw phase-shift values for both wavelengths, 684 nm (light blue line) and 828 nm (dark blue line), are shown in Fig. 3b. The increase in the phase shift of the 828 nm wavelength results in the spread of the cerebral saturation centiles of approximately 20% saturation, and the changes of the phase over time result in a movement through the centiles (Fig. 3a). The visible drop in cerebral saturation is due to a small movement of the sensor during the measurement. The phase-shift values of the single measurement are comparable to the overall phase shift of the whole FD-NIRS group, 684 nm 6.23 [6.04–6.43], 828 nm 7.70 [7.22–8.07] and 684 nm 6.04 [4.46–6.54], 828 nm 7.20 [5.66–7.81], respectively. The median phase-shift values of all included FD-NIRS measurements are presented in supplementary Table I.

### SpO_2_

Regarding the whole study population, the postductal SpO_2_ values increased during the transition period (Fig. 2c and d). The SpO_2_ values were significantly different between neonates with and without the need for respiratory support (*p* < 0.01) and between the CW-NIRS and FD-NIRS groups (*p* < 0.01). SpO_2_ values in the first four minutes after birth were predominantly lower in the FD-NIRS group.

### Haemoglobin concentration

**Fig. 4:**
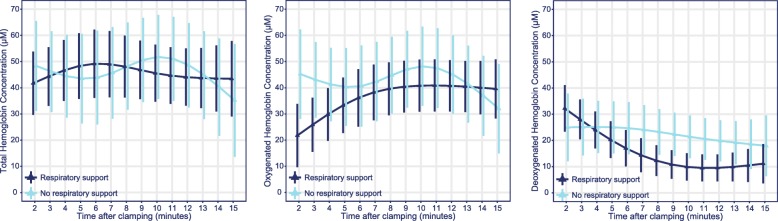
Effect plots of haemoglobin concentration measurements. **a**, Total haemoglobin concentration (THb); **b**, oxygenated haemoglobin concentration (O_2_Hb); and **c**, deoxygenated haemoglobin concentration (HHb). THb, O_2_Hb and HHb are presented as per μM. Symbols represent the estimated means with associated 95% confidence intervals for all difference time points, based on a mixed model including (non-linear) time, respiratory support and their interaction

The course of THb over the 15-min window did not significantly differ between neonates with and without respiratory support (*p* = 0.45); the same holds true for O_2_Hb (*p* = 0.20) and HHb (*p* = 0.10). The effect plots of the estimated means (Fig. 4) show a fairly constant THb value and a decrease over time for HHb. For O_2_Hb, an increase over time is seen in the respiratory support group, but such an increase is less evident in the non-respiratory support group. A significant time-dependent trend was seen for HHb values (*p* < 0.01) but not for THb values (*p* = 0.45). The time-dependent trend of O_2_Hb did not reach statistical significance (*p* = 0.08).

## Discussion

We compared the outcomes of two different measurement devices of cerebral oxygenation, INVOS 5100C (CW-NIRS) and OxiplexTS™ (FD-NIRS), during the immediate transition after birth in preterm neonates. After adjustment for the need for respiratory support, the time-dependent trend of the increase in cerebral oxygenation measured with the FD-NIRS device was significantly different from that measured with the CW-NIRS device. Not only the onset but also the slope differed over time. The need for respiratory support significantly influenced the course of the increase in cerebral saturation, irrespective of the device used.

From a technical perspective, the FD-NIRS device is superior. Clinically, we expect to see low cerebral saturation values at the beginning of the transition after birth. However, this is largely because most literature on cerebral saturation during the transition after birth is based on low saturation measurements with the INVOS. The lack of a gold-standard technique for the measurement cerebral saturation during this period makes it difficult to assess if these values are correct. From a medical point of view, we expect the SpO_2_ values to be higher than the measured rcSO_2_ values, which is not the case for the FD-NIRS group. This may indicate that FD-NIRS overestimates cerebral saturation.

The phase-shift values of 684 nm remain fairly constant throughout the single measurement, whereas the phase-shift values of 828 nm start at roughly the same point as 684 nm but increase over time. The magnitude of the change in the phase shift directly influences the spread between the centiles of the recalculated cerebral saturations in Fig. 3. Supplementary Table I shows that the presented measurement is comparable to that of most other patients measured with FD-NIRS in this study. The changes in the phase shift over time are likely to improve the accuracy of an FD-NIRS device but limit the comparability between the FD-NIRS device and the CW-NIRS device. Without the knowledge of which physiological parameters are changing when and by how much, or of how they influence the phase measurement over time, and without more research into what other influences on the phase measurement exist, it is impossible to determine if phase-shift correction improves the cerebral saturation measurement. What is clear is that without it, the FD-NIRS measurements could change by as much as 20% rcSO_2_.

In both centres, immediate cord clamping was the standard of care. Although not significant, delayed cord clamping (DCC; > 60 s) has been shown to be associated with lower rcSO_2_ immediately after cord clamping but with higher values when stabilized than immediate cord clamping [11]. Additionally, a recent study concluded that DCC ≥ 30 s was associated with improved cerebral oxygenation in the first 24 h after birth [12]. Although immediate cord clamping was the standard of care, an average delay in cord clamping in one of our centres or between infants with and without the need for respiratory support may have contributed to the differences found between devices. Medical professionals should be aware that DCC affects cerebral saturation immediately after birth and may lead to different onset of and increase in rcSO_2_.

Several studies comparing different devices, sensors and measurement techniques showed differences up to 14% between rcSO_2_ values [13–17]. One of these studies made use of stepwise induced hypoxia in healthy volunteers [16]. In line with our findings, measurements of brain-derived parameters in that study significantly differed between FD-NIRS and CW-NIRS measurement techniques. FD-NIRS was not considered advantageous in parameter recovery [16]. The within-subject reproducibility of cerebral oxygenation measurements can differ up to 10% [14, 17–19]. Reproducibility, however, is of less importance for trend monitoring. To identify deviations from normal cerebral oxygenation, quantitative monitoring is key [20]. Previous research using FD-NIRS to evaluate infants’ brain development showed consistent results from repeated measurements [21, 22]. In 2007, the European Society for Paediatric Research proposed increasing the validity and comparability of peripheral NIRS measurements by standardizing the approach [23]. This important initiative has to be extended to standardizing cerebral NIRS measurements, supported by the possibility of converting values from one oximeter to another. A first valuable attempt has been made using in vitro phantom testing [24, 25]. Furthermore, we recommend that the algorithms used by NIRS devices be published, as this enables us to investigate and understand where the differences in the readings come from.

The SpO_2_ values of neonates requiring respiratory support were slightly lower than those of neonates who did not require respiratory support. This finding may be due to inadequate lung aeration in neonates requiring respiratory support. Previous studies have reported both lower SpO_2_ and cerebral oxygen saturation values in neonates requiring respiratory support [7, 26]. In the present study, the trend in the postductally measured SpO_2_ values differed between the FD-NIRS and CW-NIRS groups, with predominantly lower values in the FD-NIRS group. Cerebral oxygenation is not solely determined by SpO_2_, but the higher rcSO_2_ values in the FD-NIRS group would likely have been even higher if the SpO_2_ would have been the same for both groups. The difference in SpO_2_ values between the groups makes proper comparison of the absolute differences between measured rcSO_2_ values nearly impossible.

The observed THb was stable over the 15-min monitoring period. As changes in arterial haemoglobin concentration are negligible, a clear decrease or increase in cerebral blood volume is not evident from these data, in contrast to the findings of Schwaberger et al. [27]. For all three FD-NIRS parameters, there were no significant differences between neonates with and without the need for respiratory support. Although not significant, the expected increase over time for O_2_Hb occurred in the respiratory support group but not in the group of neonates who did not receive respiratory support, where even a slight decrease was seen. A possible explanation is the low number of measurements in the latter group, as infants were transferred to the resuscitation table after a few minutes with the parents, motion artefacts occurred and infants not needing respiratory support were more likely to return to the mother before 15 min as they were considered stable.

The CW-NIRS technique and the FD-NIRS technique each have shortcomings. By using a continuous light source, CW-NIRS assumes a degree of scattering. Due to the modulation of the emitted light, FD-NIRS allows for the quantification of the amount of light scatter, which theoretically results in more accurate measurements of tissue oxygenation. Moreover, FD-NIRS provides high-frequency raw data, resulting in a ‘noisier’ output. In addition, the device is equipped with a reusable but delicate and cumbersome neonatal sensor that easily results in motion artefacts due to movements of the infant and limits bedside usability and patient comfort. In addition, the OxiplexTS™ does not have a CE certificate for clinical use; therefore, it is available only for research purposes. For both devices, tissue homogeneity is assumed, which is debated in regard to the neonatal brain [22, 28]. In an inhomogeneous structure, the use of the mean pathlength by the FD-NIRS may overestimate or underestimate the absolute values of oxygenation.

Several limitations of this study need to be addressed. First, this study compared two devices incorporating different measurement techniques. The observed differences in this study may not be solely attributable to differences between FD-NIRS and CW-NIRS. The differences may result from differences between devices (e.g., different algorithms and emitter-detector differences), as discrepancies between devices using the same measurement technique are reported [13–15, 24, 25]. As this study was a comparison between two specific NIRS devices, the results may not be representative of the whole spectrum of available CW-NIRS and FD-NIRS devices.

Second, FD-NIRS measurement data from our centre were compared with CW-NIRS data from the centre in Graz [8, 29]. Although individual patient data were used, allowing for repeated measurement comparisons using mixed models, a randomized controlled trial would have been preferable. Third, while all included children in Graz were born by caesarean section, the included children in our centre were born either by vaginal delivery or by caesarean section. Nevertheless, previous research showed no differences in rcSO_2_ with respect to the mode of delivery, nor did our data [30]. Fourth, although not of significant influence on our model, the difference in gestational age between the FD-NIRS and CW-NIRS groups may have caused differences in measured rcSO_2_ and SpO_2_ values and may have affected the need for respiratory support. Fifth, the SpO_2_ was measured postductally, as preductal measurements could not be recorded without interfering with routine medical care in the FD-NIRS patients. As postductal SpO_2_ values tend to be lower than preductal SpO_2_ values, fractional tissue oxygen extraction values were not calculated.

## Conclusion

This research demonstrated that rcSO_2_ values and trends over time differed between devices, mostly in the first minutes after birth. It remains challenging to compare results from different devices, as not only the specific technique but also the specific algorithms and emitter-detector distances may have had a large impact on the outcomes. The FD-NIRS technique might be technically superior to the CW-NIRS technique, but from a clinical point of view, the FD-NIRS technique seems to overestimate true cerebral oxygenation. To date, in the neonatal transition period, the technical superiority of FD-NIRS devices does not outweigh the usability of the more clinically viable and widely used CW-NIRS devices. Additional haemoglobin concentration measurements may, in a research setting, provide more information on changes in cerebral haemodynamics. The absolute differences found may not be relevant in clinical practice, as trend monitoring of rcSO_2_ in combination with SpO_2_ may suffice to guide support during the transition.

## Supplementary information

**Additional file 1.** Table SI: Phase shift of the patients measured with FD-NIRS

## Data Availability

The data that support the findings of this study are available from Erasmus MC - Sophia Children’s Hospital, but restrictions apply to the availability of data from the Medical University of Graz, which were used under licence for the current study, and so are not publicly available. Data are, however, available from the authors upon reasonable request and with permission from the Medical University of Graz.

## Abbreviations

AC: Amplitude of modulation
CW: Continuous-wave
DC: Average intensity
FD: Frequency-domain
HHb: Deoxygenated haemoglobin concentration
HR: Heart rate
NIRS: Near-infrared spectroscopy
O_2_Hb: Oxygenated haemoglobin concentration
PEEP: Positive end-expiratory pressure
rcSO_2_: Regional cerebral oxygen saturation
SpO_2_: Arterial oxygen saturation
THb: Total haemoglobin concentration

## References

[1] Pansy J, Baik N, Schwaberger B, Scheuchenegger A, Pichler-Stachl E, Avian A (2017). Cerebral hypoxia during immediate transition after birth and short term neurological outcome. Early Hum Dev.

[2] Verhagen EA, Van Braeckel KN, van der Veere CN, Groen H, Dijk PH, Hulzebos CV (2015). Cerebral oxygenation is associated with neurodevelopmental outcome of preterm children at age 2 to 3 years. Dev Med Child Neurol.

[3] Viaroli F, Cheung PY, O'Reilly M, Polglase GR, Pichler G, Schmolzer GM (2018). Reducing Brain Injury of Preterm Infants in the Delivery Room. Front Pediatr.

[4] Garvey AA, Kooi EMW, Smith A, Dempsey EM. Interpretation of Cerebral Oxygenation Changes in the Preterm Infant. Child (Basel). 2018;5(7). 10.3390/children5070094 PMC6069134 29987227

[5] Schneider A, Minnich B, Hofstatter E, Weisser C, Hattinger-Jurgenssen E, Wald M (2014). Comparison of four near-infrared spectroscopy devices shows that they are only suitable for monitoring cerebral oxygenation trends in preterm infants. Acta Paediatr.

[6] Hessel TW, Hyttel-Sorensen S, Greisen G (2014). Cerebral oxygenation after birth - a comparison of INVOS((R)) and FORE-SIGHT near-infrared spectroscopy oximeters. Acta Paediatr.

[7] Binder C, Urlesberger B, Avian A, Pocivalnik M, Muller W, Pichler G (2013). Cerebral and peripheral regional oxygen saturation during postnatal transition in preterm neonates. J Pediatr.

[8] Pichler G, Binder C, Avian A, Beckenbach E, Schmolzer GM, Urlesberger B (2013). Reference ranges for regional cerebral tissue oxygen saturation and fractional oxygen extraction in neonates during immediate transition after birth. J Pediatr.

[9] R Core Team. R: A language and environment for statistical computing. R Foundation for Statistical Computing. Vienna, Austria2017.

[10] Fenton TR, Kim JH (2013). A systematic review and meta-analysis to revise the Fenton growth chart for preterm infants. BMC Pediatr.

[11] Pichler G, Baik N, Urlesberger B, Cheung PY, Aziz K, Avian A (2016). Cord clamping time in spontaneously breathing preterm neonates in the first minutes after birth: impact on cerebral oxygenation - a prospective observational study. J Matern Fetal Neonatal Med.

[12] Popat H, Galea C, Evans N, Lingwood B, Colditz PB, Halliday R (2019). Effect of delayed cord clamping on cerebral oxygenation in very preterm infants. Neonatology..

[13] Dix LM, van Bel F, Baerts W, Lemmers PM (2013). Comparing near-infrared spectroscopy devices and their sensors for monitoring regional cerebral oxygen saturation in the neonate. Pediatr Res.

[14] Pocivalnik M, Pichler G, Zotter H, Tax N, Muller W, Urlesberger B (2011). Regional tissue oxygen saturation: comparability and reproducibility of different devices. J Biomed Opt.

[15] Szczapa T, Karpinski L, Moczko J, Weindling M, Kornacka A, Wroblewska K (2013). Comparison of cerebral tissue oxygenation values in full term and preterm newborns by the simultaneous use of two near-infrared spectroscopy devices: an absolute and a relative trending oximeter. J Biomed Opt.

[16] Davies DJ, Clancy M, Lighter D, Balanos GM, Lucas SJE, Dehghani H (2017). Frequency-domain vs continuous-wave near-infrared spectroscopy devices: a comparison of clinically viable monitors in controlled hypoxia. J Clin Monit Comput.

[17] Dullenkopf A, Kolarova A, Schulz G, Frey B, Baenziger O, Weiss M (2005). Reproducibility of cerebral oxygenation measurement in neonates and infants in the clinical setting using the NIRO 300 oximeter. Pediatr Crit Care Med.

[18] Menke J, Voss U, Moller G, Jorch G (2003). Reproducibility of cerebral near infrared spectroscopy in neonates. Biol Neonate.

[19] Sorensen LC, Greisen G (2006). Precision of measurement of cerebral tissue oxygenation index using near-infrared spectroscopy in preterm neonates. J Biomed Opt.

[20] Scheeren TW, Schober P, Schwarte LA (2012). Monitoring tissue oxygenation by near infrared spectroscopy (NIRS): background and current applications. J Clin Monit Comput.

[21] Franceschini MA, Thaker S, Themelis G, Krishnamoorthy KK, Bortfeld H, Diamond SG (2007). Assessment of infant brain development with frequency-domain near-infrared spectroscopy. Pediatr Res.

[22] Zhao J, Ding HS, Hou XL, Zhou CL, Chance B (2005). In vivo determination of the optical properties of infant brain using frequency-domain near-infrared spectroscopy. J Biomed Opt.

[23] Pichler G, Wolf M, Roll C, Weindling MA, Greisen G, Wardle SP (2008). Recommendations to increase the validity and comparability of peripheral measurements by near infrared spectroscopy in neonates. 'Round table', section of haematology, oxygen transport and microcirculation, 48th annual meeting of ESPR, Prague 2007. Neonatology.

[24] Kleiser S, Nasseri N, Andresen B, Greisen G, Wolf M (2016). Comparison of tissue oximeters on a liquid phantom with adjustable optical properties. Biomed Opt Express..

[25] Kleiser S, Ostojic D, Andresen B, Nasseri N, Isler H, Scholkmann F (2018). Comparison of tissue oximeters on a liquid phantom with adjustable optical properties: an extension. Biomed Opt Express.

[26] Schwaberger B, Pichler G, Binder C, Avian A, Pocivalnik M, Urlesberger B (2014). Even mild respiratory distress alters tissue oxygenation significantly in preterm infants during neonatal transition. Physiol Meas.

[27] Schwaberger B, Pichler G, Binder-Heschl C, Baik N, Avian A, Urlesberger B (2015). Transitional changes in cerebral blood volume at birth. Neonatology..

[28] Arri SJ, Muehlemann T, Biallas M, Bucher HU, Wolf M (2011). Precision of cerebral oxygenation and hemoglobin concentration measurements in neonates measured by near-infrared spectroscopy. J Biomed Opt.

[29] Baik N, Urlesberger B, Schwaberger B, Schmolzer GM, Mileder L, Avian A (2015). Reference ranges for cerebral tissue oxygen saturation index in term neonates during immediate neonatal transition after birth. Neonatology..

[30] Urlesberger B, Kratky E, Rehak T, Pocivalnik M, Avian A, Czihak J (2011). Regional oxygen saturation of the brain during birth transition of term infants: comparison between elective cesarean and vaginal deliveries. J Pediatr.

