# Supplementary material for: Comparison of frequency-domain and continuous-wave near-infrared spectroscopy devices during the immediate transition
Source: BMC Pediatr. 2020 Feb 28;20:94. doi: 10.1186/s12887-020-1987-4 (PMC7047398; doi:10.1186/s12887-020-1987-4)
Supplement: Supplementary file 1 — Additional file 1. Table SI: Phase shift of the patients measured with FD-NIRS [file 12887_2020_1987_MOESM1_ESM.docx]

| *Supplementary table I: Phase shift of the patients measured with FD-NIRS* | | |
| --- | --- | --- |
| *Patient* | *Phase 684nm* | *Phase 828nm* |
| *1* | *2.97 [2.48 – 3.46]* | *3.10 [2.59 – 3.47]* |
| *2* | *4.60 [3.98 – 4.92]* | *5.17 [4.38 – 5.73]* |
| *3* | *3.78 [3.64 – 3.93]* | *2.59 [2.41 – 2.80]* |
| *4* | *6.57 [6.04 – 6.99]* | *8.14 [7.40 – 9.14]* |
| *5* | *6.79 [6.30 – 7.34]* | *7.45 [7.14 – 7.95]* |
| *6* | *8.81 [8.60 – 9.16]* | *10.91 [10.63 – 11.05]* |
| *7* | *6.59 [5.77 – 7.68]* | *5.81 [4.94 – 6.96]* |
| *8* | *5.10 [4.78 – 5.48]* | *7.10 [6.54 – 7.53]* |
| *9* | *6.54 [6.27 – 6.97]* | *6.43 [5.58 – 7.01]* |
| *10* | *6.39 [6.29 – 6.57]* | *7.06 [6.84 – 7.39]* |
| *11* | *4.82 [4.62 – 5.02]* | *-13.10 [-13.30 - -12.87]* |
| *12* | *2.21 [1.73 – 2.62]* | *3.54 [2.74 – 3.97]* |
| *13* | *6.23 [6.04 – 6.43]* | *7.70 [7.22 – 8.07]* |
| *14* | *6.41 [4.87 – 7.07]* | *7.28 [5.62 – 8.48]* |
| *15* | *7.79 [7.35 – 8.05]* | *9.50 [8.53 – 10.48]* |
| *16* | *6.33 [6.07 – 6.71]* | *7.66 [6.91 – 8.19]* |
| *17* | *9.52 [8.93 – 9.87]* | *48.32 [46.22 – 49.29]* |
| *18* | *6.33 [5.82 – 6.91]* | *8.12 [6.75 – 8.94]* |
| *19* | *8.14 [7.75 – 8.89]* | *9.06 [7.62 – 10.11]* |
| *20* | *6.34 [5.54 – 6.73]* | *7.68 [5.39 – 8.84]* |
| *21* | *3.36 [3.27 – 3.90]* | *5.42 [4.63 – 5.83]* |
| *22* | *4.53 [4.32 – 4.68]* | *6.43 [4.66 – 7.74]* |
| *23* | *6.93 [6.07 – 8.67]* | *8.86 [8.08 – 9.51]* |
| *24* | *4.06 [3.66 – 4.41]* | *6.70 [6.17 – 7.07]* |
| *25* | *5.54 [5.35 – 5.77]* | *7.47 [7.15 – 7.90]* |
| *26* | *6.49 [5.69 – 7.55]* | *7.59 [6.99 – 8.33]* |
| *27* | *5.48 [4.92 – 6.05]* | *5.48 [4.61 – 6.41]* |
| *28* | *3.45 [3.25 – 5.06]* | *4.70 [4.47 – 5.37]* |
| *29* | *6.53 [5.29 – 8.46]* | *7.98 [6.96 – 8.52]* |
| *30* | *4.44 [3.82 – 5.08]* | *5.66 [4.86 – 6.45]* |
| *31* | *6.93 [6.17 – 7.35]* | *9.05 [8.22 – 9.56]* |
| *32* | *4.30 [3.65 – 4.83]* | *5.68 [5.23 – 6.06]* |
| *33* | *5.93 [5.80 – 6.05]* | *7.72 [7.30 – 7.89]* |
| *34* | *5.89 [5.05 – 7.10]* | *5.88 [5.39 – 6.36]* |
| *35* | *4.16 [3.80 – 4.43]* | *5.85 [5.10 – 6.87]* |
| *36* | *6.15 [5.48 – 6.42]* | *7.84 [6.90 – 8.95]* |
| *37* | *3.27 [2.22 – 5.12]* | *2.55 [1.35 – 5.73]* |
| *38* | *5.37 [4.41 – 5.76]* | *7.58 [5.39 – 8.81]* |
| Values are median [interquartile range] | | |
